# Label-free (fluorescence-free) sensing of a single DNA molecule on DNA origami using a plasmon-enhanced WGM sensor

**DOI:** 10.1515/nanoph-2024-0560

**Published:** 2025-01-20

**Authors:** Shahin Ghamari, Germán Chiarelli, Karol Kołątaj, Sivaraman Subramanian, Guillermo P. Acuna, Frank Vollmer

**Affiliations:** Department of Physics and Astronomy, Living Systems Institute, University of Exeter, Exeter, EX4 4QD, UK; Department of Physics, University of Fribourg, Chemin du Musée 3, Fribourg CH-1700, Switzerland; Swiss National Center for Competence in Research (NCCR) Bio-Inspired Materials, University of Fribourg, Chemin des Verdiers 4, CH-1700 Fribourg, Switzerland

**Keywords:** single-molecule techniques, DNA origami, whispering gallery modes, optoplasmonic, DNA-PAINT

## Abstract

The integration of DNA origami structures with opto-plasmonic whispering gallery mode (WGM) sensors offers a significant advancement in label-free biosensing, overcoming the limitations of traditional fluorescence-based techniques, and providing enhanced sensitivity and specificity for detecting DNA hybridization events. In this study, DNA origami acts as a scaffold for the precise assembly of plasmonic dimers, composed of gold nanorods (AuNRs), which amplify detection sensitivity by generating strong near-field enhancements in the nanogap between the nanorods. By leveraging the strong electromagnetic fields generated within the nanogap of the plasmonic dimer, this platform enables the detection of transient hybridization events between DNA docking strands and freely diffusing complementary sequences. Our findings demonstrate that the salt concentration critically influences DNA hybridization kinetics. Higher ionic strengths reduce electrostatic repulsion between negatively charged DNA strands, thereby stabilizing duplex formation and prolonging interaction times. These effects are most pronounced at salt concentrations around 300–500 mM, where optimal conditions for duplex stability and reduced dissociation rates are achieved. By thoroughly investigating the hybridization kinetics under varying environmental conditions, this study contributes to a deeper understanding of DNA interactions and offers a robust tool for single-molecule detection with real-time capabilities.

## 1 Introduction

Over the past decades, the rapid advancement of nanotechnology has spurred the development of innovative sensing platforms for detecting biomolecules without the need for amplification [1], [2], [3]. Among other enabling techniques, DNA nanotechnology stands out due to its flexibility and programmability that takes advantage of the specificity of DNA base pairing, which enables the creation of highly precise structures for sensing using the DNA origami technique [4]. This method has been extensively explored to self-assemble often complex shapes with high degree of flexibility and dimensions in the hundreds of nm such as quasi 2D and 3D geometries [5], [6], [7], [8]. In addition, these structures can be exploited as nano-breadboards to host hybrid species such as proteins [9], metallic and high-index nanoparticles [10], [11], [12], mRNAs [13] single photon emitters including organic fluorophores and quantum dots [14], carbon nanotubes and biological materials, into intricate geometries with nanometer precision and stoichiometric control [15], [16], [17], [18], [19], [20]. Naturally, these DNA origami structures proved to be highly valuable for developing sensor platforms, particularly in the field of biosensing [21], [22], [23], [24]. To date, DNA origami structures are consolidated as robust platforms for molecular sensing, that can enhance the detection of signals arising from single molecules through the assembly of metallic nanoparticles forming optical antennas [25], [26]. Most efforts in this direction involve fluorescence labelling [22] or alternatively, the detection of DNA strands can be performed through for example measurement of chiral properties [27], [28]. However, these approaches can encounter challenges when analyte oligonucleotides either cannot be efficiently labelled or when the photodecomposition of fluorophores complicates analysis. Furthermore, DNA sensing by chiral measurements requires often complex instrumentation [29]. This limitation has prompted researchers to seek alternative approaches, leading to the exploration of fluorescence-free sensor modalities, commonly referred to as ‘label-free’ methods that do not rely on chiral measurements.

Label-free methods, such as the integration of whispering gallery mode (WGM) sensors and plasmonic particles (opto-plasmonic WGM sensor), exploit the modification of the plasmonic resonance upon changes on the particle surface such as the binding of specific analytes and can offer significant advantages by avoiding challenges associated with fluorescent labelling. The local enhancement of optical near-fields around the plasmonic particles allows these label-free sensors to exhibit exceptionally high detection sensitivity, even for small biomolecules like short DNA oligonucleotides [30], [31]. Typically, this approach is implemented by functionalizing the surface of metallic nanoparticles with different functional groups such as DNA oligonucleotides or proteins using thiol modifications that exhibit a high affinity to coinage metals. As a result, this method has the following limitations. First, the analytes can bind in principle anywhere on the nanoparticle surface, however detection will be more sensitive at the hotspots of plasmonic near-field enhancements. Second, with this approach it is extremely challenging to create geometries of nanoparticles such as dimers that would lead to stronger hotspots. Finally, it is difficult to multiplex detection to address different analytes at the same time. In order to circumvent these limitations, the DNA origami technique appears as an ideal candidate as it can arrange different nanoparticles in complex geometries, and it allows the use of different, orthogonal (non-interfering) DNA sequences for multiplexed detection precisely at the hotspot and can thus lead to more precise and reliable measurements overcoming limitations seen in traditional methods where the same DNA strands serve both purposes.

In this study, we introduce an approach for label-free sensing of a single DNA molecule on a DNA origami structure utilizing the opto-plasmonic WGM sensor based on a glass microsphere. The DNA origami structure is used as a precisely engineered platform for assembling a plasmonic dimer based on two AuNRs. By harnessing the near-field enhancements within the nanogap of the plasmonic dimer, this sensor can effectively detect specific single-molecule transient hybridization events, between DNA docking strands located within the nanogap assembled on the DNA origami and freely diffusing target complementary sequences, thereby increasing the sensitivity and reducing the sensing area.

## 2 Opto-plasmonic WGM setup

**Figure 1:**
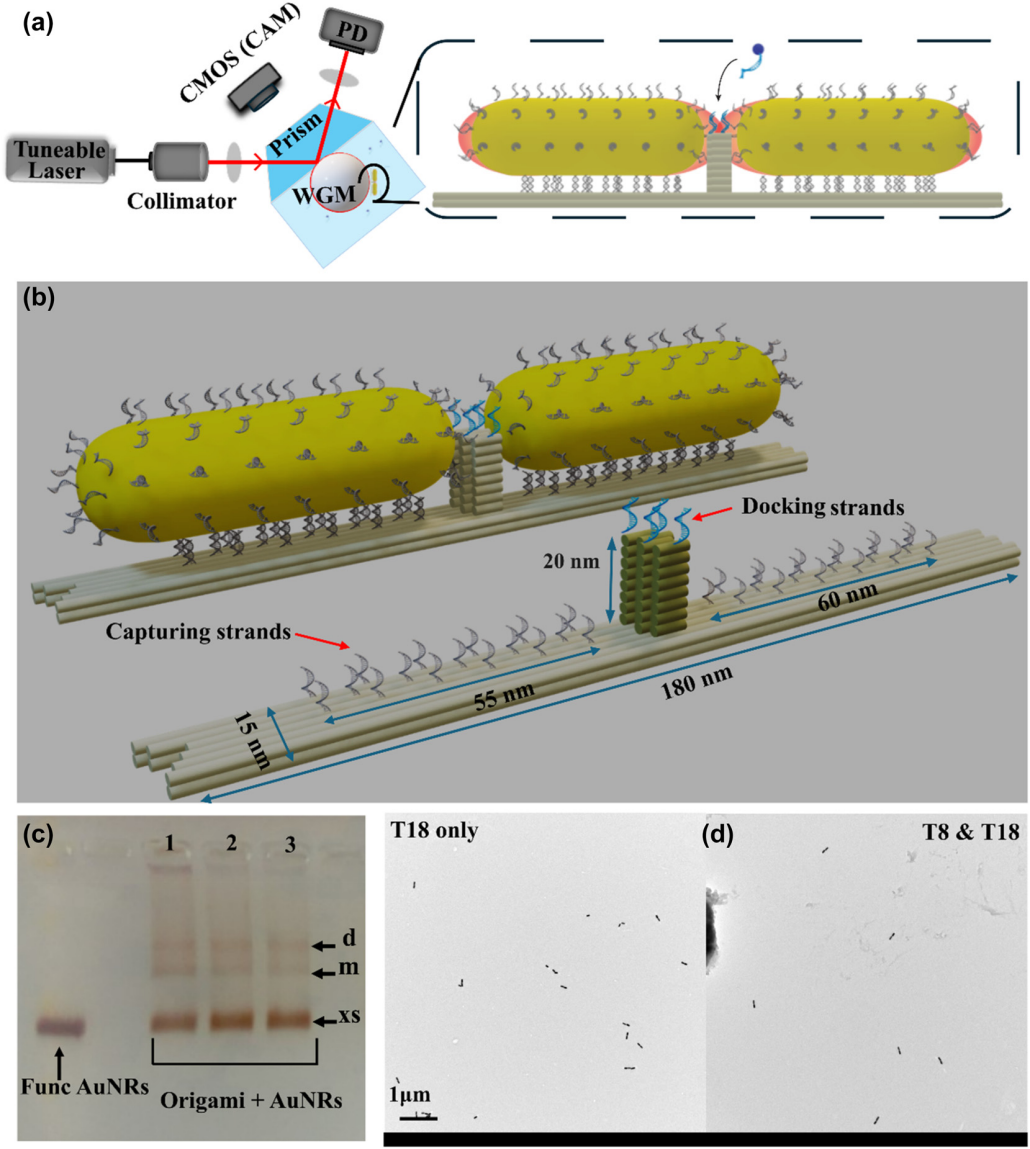
Schematic of sensing platforms. (a) WGM-based sensing set-up. The excitation laser beam focuses on the surface of the prism to excite the WGM modes in a spherical glass microcavity. Following this, gold nanorod dimers are immobilized onto the surface of the salinized microsphere. The zoomed-in view shows the plasmonic DNA origami-assembled nanorod dimer, with capturing strand in grey used to bind nanorods to the origami structure and docking strands in blue with different sequence located at the hotspot used for transient binding with freely diffusive complementary strands in blue. (b)–(d) DNA origami nano-antennas design and characterization. (b) A perspective of the DNA origami structure, highlighting the 35 poly-A extensions in grey and the 4 docking strands located on the top of the mast marked in blue. This design provides the framework for incorporating the AuNRs in a tip-to-tip configuration. (c) Gel electrophoresis image displaying the bands resulting from the DNA origami and AuNR mixture after the annealing ramp. The first column serves as a reference and contains only functionalized AuNRs (Func AuNRs). The subsequent columns represent bands corresponding to similar syntheses, repeated three times for consistency. The lowermost band corresponds to the excess of AuNRs (xs), while the bands above it represent the monomer (m) and dimer (d) structures, from down to up order. Additional bands above the dimer band may appear, indicating larger structures containing more than two AuNRs. (d) The left image displays the structures formed using only T18 strands, yielding a lower percentage of well-aligned dimers (about 60 %). In contrast, the right image shows the improved alignment and yield (approximately 90 %) of dimers achieved by using T8 and T18 strands.

The schematic in Figure 1(a) shows the experimental setup used in this work. It consists of a custom-built prism optical microscope based on an external cavity diode laser, which operates with a scan rate of 50 Hz (Toptica TA pro 780HP, Toptica GmbH, Munich, Germany). Light is focused onto the back surface of a high refractive index prism made of N-SF11 glass (*n*_780nm_ ≈ 1.77), whereas on the other surface, a glass microsphere with a diameter of approximately 90 μm (for more details on microsphere fabrication, see protocol in SI1) is positioned. Whispering gallery modes in the glass microsphere, are excited through frustrated total internal reflection (FTIR) at the prism’s surface [32]. Real-time imaging of the laser spot, the microcavity, and the alignment of the resonator with respect to the prism for efficient coupling is achieved using an imaging system consisting of an Olympus 10× objective with an *f* = 200 mm objective lens (AC254-200-B, Thorlabs GmbH, Germany), and a CMOS camera (DCC1545M, Thorlabs GmbH, Germany). A photodetector (PD) (PDA10AZ-EC, Thorlabs Inc.) is employed to collect the reflected light off the prism surface within the output arm of the setup. For convenient sample injection and manual chamber rinsing, a v-shaped sample chamber made of polydimethylsiloxane (PDMS) with a volume of 200–300 μl is sandwiched between the prism and a microscope cover glass slide. All experiments were conducted at a temperature of 22 °C.

## 3 Synthesis and characterization of the DNA origami nano-antennas

The DNA origami structure utilized in this study is based on a rectangular design [33] with a “mast”, which is about 20 nm in height, at the center (see Figure 1(b)). To incorporate previously functionalized AuNRs, the DNA origami was modified by adding a total of 17 and 18 capturing strands (CSs) at the left and right side of the structure, respectively (see S2A). These CSs were composed of 8-nucleotide long single-stranded DNA (ssDNA) extensions consisting exclusively of adenine (poly-A), designed to form stable permanent hybridization with the poly-T ssDNA on the functionalized AuNRs. The position and length of the CSs resulted from a parameter optimization to ensure the correct orientation of the rods along the origami and to prevent perpendicular alignment. The AuNRs used had nominal dimensions of 25 nm in diameter and 75 nm in length, with corresponding nominal surface plasmon resonance peaks at 514 nm (transverse mode) and 700 nm (longitudinal mode). Initially, the AuNRs were functionalized using 18-nucleotide long ssDNA of only thymine (T18), through the freeze-thaw method [34]. However, during our investigation, we found that modifying the AuNRs functionalization protocol by introducing a 4:1 ratio of thiolated poly-T shorter strands (8 nucleotides in length) to the standard 18-long poly-T strands significantly improved the yield of well-aligned dimers from approximately 60 % using only T18 strands to around 90 %. Once the origami and AuNRs were prepared, they were mixed at a ratio of 1:10 using in particular 20 µl of a solution containing the DNA origami at a concentration of 2 nM. To mitigate DNA electrostatic repulsion, NaCl was added to a final concentration of 300 nM. The mixture was annealed in a two-ramps process, initially heated to 45 °C and then gradually cooled down to 20 °C at a rate of 30 min per degree. It was subsequently reheated to 35 °C before being gradually cooled back to 20 °C using a similar ramping procedure. The synthesized structures were then purified by electrophoresis using a 1 % agarose gel in a 1× TAE 12 mM MgCl_2_ buffer at 70 V for 180 min, while being cooled in an ice water bath. Figure 1(c) presents an example of a gel obtained after the electrophoresis process. The first column serves as a reference, containing functionalized AuNRs without DNA origami (Func AuNRs). Each subsequent column (labelled 1, 2 and 3) represents a synthesis of the desired antenna structure. The bottommost band corresponds to the expected excess of Func AuNRs. Above it, two distinct sharper bands can be observed, representing the monomer (lower band) and dimer (upper band) structures. Upon identifying the dimer band, it was carefully extracted from the gel and gently squeezed using a parafilm-covered glass slide, allowing the structures to be released from the gel matrix. These purified dimer structures are now ready for further characterization and evaluation in our label-free localized surface plasmon resonance (LSPR) sensing experiments. To study the final product of our synthesis, we used a transmission electron microscope (TEM). Figure 1(d), along with its large-scale version provided in S3, shows an example of the dimers extracted from the gel, with a 90 % yield (right image) compared to other structures observed in the TEM images (left image). The UV spectra of the functionalized AuNRs, monomers, and dimers, shown in Figure S4, illustrate the extinction coefficient measured in ensemble at low concentrations using a micro-volume spectrophotometer (Nanodrop). The AuNRs used in this study have dimensions of 25 nm in diameter and 75 nm in length. These dimensions were chosen to align the longitudinal plasmon resonance of the AuNRs with the operational wavelength of our setup. The longitudinal plasmon resonance of pure AuNRs is located at 700 nm (dotted line). When a single AuNR is attached to the origami, the resonance shifts to approximately 740 nm (monomer), while the peak for dimers is located around 760 nm, closely matching the 780 nm emission of the laser beam used for exciting WGMs. The shift in the resonance peak of approximately 20 nm from monomers to dimers is in excellent agreement with previous measurements and simulations of systems with comparable dimensions [35]. On top of the mast, the DNA origami exhibits four docking strands (DSs) based on the 11-base sequence (5′–3′) TTAAATGCCCG for the detection of single ssDNA (see S2A). At the DSs position within the dimer hotspot, we simulated the electric field enhancement for the nominal dimensions of the AuNRs at a gap of 10 nm and a wavelength of 780 nm. Enhancement values between 60 and 80 were obtained. For comparison, we also include results for the monomer, which yield much lower enhancement (see S2B).

## 4 Experimental method for detection of single-molecule DNA hybridization events

**Figure 2:**
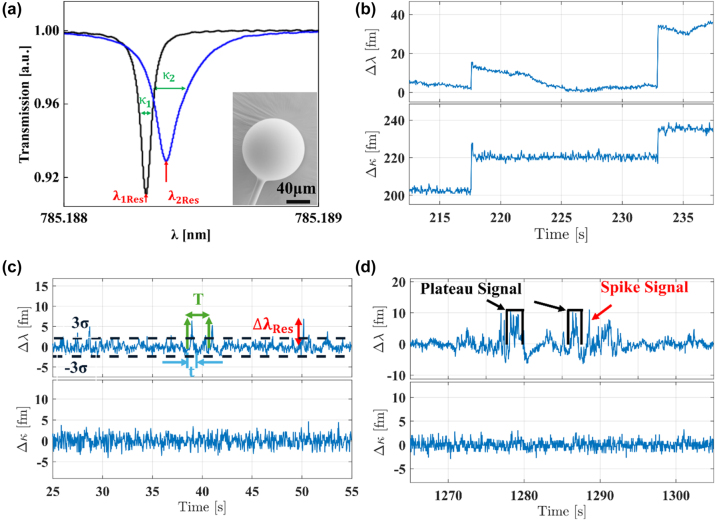
Interaction of AuNRs with the WGM sensor surface. (a) Lorentzian line shape of excited WGM position before (black) and after attachment of two dimers (blue), the shift in *λ*_1Res_ to *λ*_2Res_ and *κ*_1_ to *κ*_2_ represent this attachment, inset is SEM images of microsphere with diameter of about 114 μm. (b) Time traces of the WGM resonance position and FWHM indicating the step-like transitions due to binding of gold dimer. Because of the significant real and imaginary parts of the dimer’s polarizability both Δ*λ* and Δ*κ* experience a shift. Interaction of docking and complementary strands. (c) Time traces of WGM resonance wavelength and FWHM for interaction between docking and complementary strands with a concentration of 450 nM in water. *σ* represents the standard deviation of the background, ‘*T*’ is the arrival time between two consecutive events (dissociated waiting time), and ‘*t*’ represents event duration (bound waiting time). (d) Examples of spike and plateau-like signals were observed in a solution containing a complementary strand with a concentration of 339 nM in a 300 mM NaCl solution. The spectral and time resolution of our setup is about 1 fm and 20 ms, respectively.

To immobilize the highly negatively charged DNA origami-assembled nanorod dimer onto the surface of the microsphere requires functionalization with surfactant that carries a positive charge [36]. The process involves immersing the microsphere in a 0.5 % *v*/*v* [95:5, Ethanol:Water] (3-Aminopropyl)triethoxysilane (APTES) for approximately 50 s to modify the surface with amino groups. The sphere is then thoroughly rinsed with de-ionized (DI) water to remove any excess and unbound APTES. After rinsing, the microsphere is placed in a chamber containing 220 μl DI water. After aligning the sphere for optimal coupling [37], WGMs were excited using a laser beam with a 780 nm wavelength, achieving coupling efficiencies of 10–40 %. The coupling of light at the WGM resonance frequency manifests as Lorentzian resonance dips across a wide scan bandwidth which is measured in the transmission spectrum (see Figure 2(a)). Subsequently, a centroid fitting algorithm [38] is employed to extract the position and width of WGM resonance, *λ*_Res_ and full width at half maximum (FWHM) known as *κ* respectively.

Then, the plasmonic DNA origami-assembled nanorod dimer was introduced into the sample chamber at a final concentration of 1.3 pM. After attachment of a plasmonic DNA origami-assembled nanorod dimer to the surface of the sphere, their plasmon resonance is stimulated by the TE mode of the WGM. Figure 2(a) illustrates the fundamental WGM mode obtained before and after attachment of the dimers to the surface of the glass microsphere. The initial WGM transmission spectrum (black) undergoes shift and broadening (blue) upon the attachment of dimers. This fractional shift of the position and width of WGM resonance results from the interaction of dimers with the evanescent field of the WGM. This small shift in resonance is directly proportional to the orientation and possibly their exact distance from the resonator surface, as well as the polarizability of the dimers in the presence of electric field of WGM, as described in Ref. [39].

The binding of the gold DNA origami-assembled nanorod dimer is observed in the WGM time traces, depicted as discrete step-like transitions with no corresponding falling-edge in both Δ*λ* = *λ*_1Res_ − *λ*_2Res_ and Δ*κ* = *κ*_1_ − *κ*_2_, as seen in the Figure 2(b). Time traces of the resonance shift were scanned within a range of several picometres around the resonance wavelength and *κ* changes were recorded over time. Drift correction (i.e., detrending) was then applied to remove background noises such as thermal drift [39]. The direction of the WGM wavelength shift (notably, the steps in Δ*λ* may exhibit either positive or negative values) is contingent upon the sign of the real part of the polarizability at the WGM excitation wavelength. Whereas the steps in Δ*κ* typically manifest as positive values due to increased losses with the introduction of more dimers. The imaginary part of the polarizability accounts for losses in the metal, contributing to the broader linewidth (FWHM) of the LSPR. Therefore, attachment of more dimers on the microcavity surface results in a reduction of quality (*Q*) of WGM. In this study, 3–5 dimers, each featuring 4 docking strands, were attached to the surface of the WGM sensor, resulting in a total of 12–20 docking areas and causing a shift in Δ*κ* of approximately 200–300 fm. The sample chamber was then subjected to a triple rinse with ultra-pure DI water to prepare for subsequent experiments using the WGM-plasmonic hybrid sensor. This prepares the setup for the characterization of DNA hybridization events between ssDNA DSs positioned at the hotspot of the dimer, and the freely diffusive complementary strands. Complementary strands are 10 bases sequence (5′–3′) CGGGCATTTA. Upon the loading of the complementary ssDNA into the chamber at nanomolar concentrations, DNA hybridisation events (signals) in Δ*λ* are detected in time traces as seen in the Figure 2(c) and (d). Notably, these signals are similar in character to the noise but display a higher amplitude, exceeding 3*σ* (where *σ* represents the standard deviation of the background [38]), with signal heights quantified by Δ*λ*_Res_. The Δ*κ* of the resonance typically remains mostly unaffected, as the imaginary part of the excess polarizability for most small molecules is negligible at visible wavelengths [30], [40]. The signals manifest as spike-like or plateau-like transitions, characterized by the dissociated waiting time (*T*) and bound waiting time (*t*). But in the presence of the non-complementary strands, a 10 bases sequence (5′–3′) TATGTAGATC, no spike or plateau-like signals were observed. This control measurement, shown in S6, confirms the specificity of the DNA hybridization events. This is attributed to the fact that these strands do not bind to the docking strands located at hotspot, validating the reliability of the sensor in detecting target DNA molecules. For the conditions employed throughout this work, the hybridization events are typically not permanent. Most detected signals exhibit spike-like transitions, but the duration of these transient signals is influenced by various factors, including the DNA sequence and length, as well as environmental conditions such as ionic strength, temperature, and pH. These parameters can modulate the kinetics of the hybridization process.

## 5 Association and dissociation kinetics of DNA hybridisation events

The DNA hybridisation kinetics can be characterised by two main rates, the association on-rate $\left(\kappa_{on}\right)$ and the dissociation, off-rate $\left(\kappa_{\text{off}}\right)$ . In particular, *κ*_on_ and *κ*_off_ represent the rate at which two complementary ssDNA come together to form a hybridized dsDNA, and the rate at which the formed dsDNA denaturalizes back into individual single strands, respectively. Since DNA hybridisation events occur randomly and independently over time, the distributions of the dissociated and bound waiting times, *T* and *t* respectively, follow a Poissonian distribution. *κ*_on_ and *κ*_off_ are then directly derived from single exponential fits of the corresponding exponential distributions, known as the survivor functions *S*(*T*) and *S*(*t*), respectively. These functions, detailed in the SI4, provide valuable insights into the kinetics of DNA hybridization.

**Figure 3:**
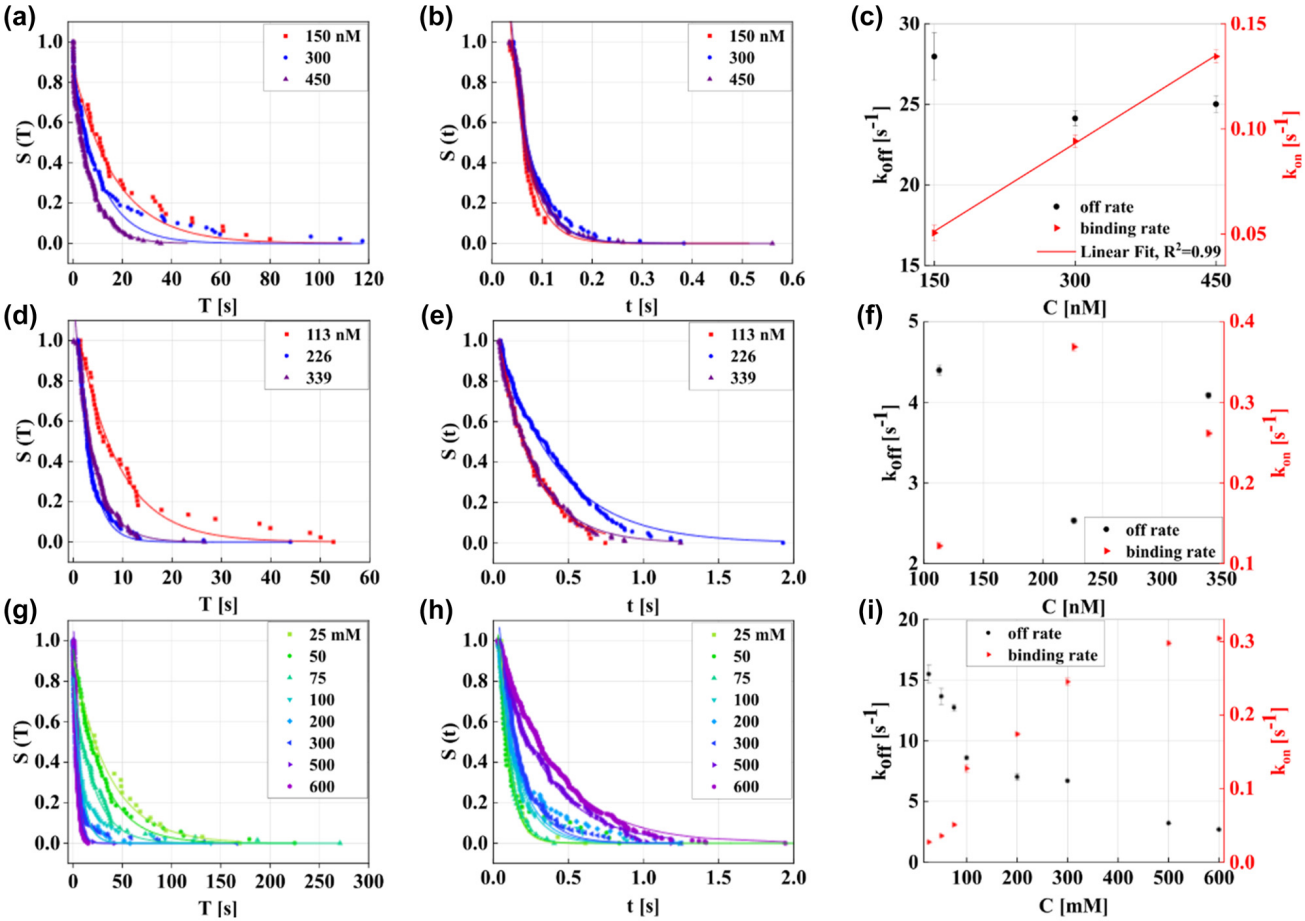
Survivor functions of dissociated and bound waiting times. (a) The survivor function of dissociated waiting time, *S*(*T*), represents the probability that no binding event has occurred within a given time interval *T* and (b) *S*(*t*), the survivor function of bound waiting time denotes the probability that a dissociation event has not occurred within an interval t. Both are shown for different complementary strand concentration (150, 300, 450 nM in red, blue and purple, respectively) in water, accompanied by their single exponential fits. (d),(e) Similarly, the survivor functions of (d) *S*(*T*) and (e) *S*(*t*) are presented for different complementary strand concentrations (113, 226, 339 nM in red, blue, and purple, respectively) in 300 mM NaCl solution, with corresponding single exponential profiles. (c),(f) Association rates (red triangles) and dissociation rates (black dots) extracted from the single exponential fit to *S*(*T*) of dissociated waiting times and *S*(*t*) of bound waiting times, respectively are depicted in (c) water and (f) 300 mM salt. The experiments in water and 300 mM NaCl are conducted over a duration of 30 min for each concentration. Graphs (g) and (h) display *S*(*T*) and *S*(*t*) for various NaCl concentrations (25, 50, 75, 100, 200, 300, 500, and 600 mM) in water, with corresponding single exponential fits. Throughout the experiments, the complementary strand concentration is maintained at 113 nM. Association rates (red triangles) and dissociation rates (black dots) are depicted in (i) for 5 different NaCl concentrations, with each concentration measured over 22 min. The on-rate and off-rate show opposite trends, where the on-rate increases from (0.03 ± 0.02) s^−1^ at 25 mM to (0.30 ± 0.05) s^−1^ at 600 mM, while the off rate decreases from (15.49 ± 0.7) s^−1^ to (2.69 ± 0.03) s^−1^. All experiments are conducted at room temperature.

In this work, the DNA hybridisation is employed as a proxy to study the concentration dependence of dissociated and bound waiting times by varying the concentration of the complementary strand in different environmental conditions. Our study also explores how different ionic concentrations influence the kinetics of DNA hybridization [41]. Sodium ions, in particular, significantly affect the stability and kinetics of DNA hybridization. These experiments provide a comprehensive understanding of how both concentration changes and variations in solution environments contribute to the overall kinetics of DNA binding and unbinding. All experiments were conducted at room temperature. Figure 3(a) and (b) illustrate the survivor probabilities of *S*(*T*) and *S*(*t*), in water for various concentrations of complementary strands. Subsequently, Figure 3(d) and (e) depict the corresponding probabilities in a 300 mM NaCl solution. The concentration of the complementary strand is incrementally increased within the chamber in steps of approximately 150 nM in water and about 113 nM in salt solution. At each concentration step, the resonance shift, Δ*λ* and Δ*κ*, are recorded for over 30 min. Figure 3(c) and (f) illustrate the event rates, association and dissociation rates, in both water and salt solutions. By single exponential fitting of survivor distribution to bound waiting times, *t*, the average values of *κ*_off_ (black dots) are obtained $\kappa_{\text{off}}^{\text{water}}=25.52\pm 0.4\;\;{\text{s}}^{-1}$ and $\kappa_{\text{off}}^{\text{salt}}=3.28\pm 0.02\;\;{\text{s}}^{-1}$ (estimate ± standard error). The *κ*_off_ values for different concentration of complementary strand remain relatively constant, consistent with findings reported in Refs. [31], [42]. The dissociation rate is notably lower in salt compared to water, suggesting prolonged interactions between complementary and docking strands in a saline environment. This effect can be related to Debye screening [38], where salt ions, especially sodium ions, shield the negatively charged phosphate backbone of DNA. This shielding reduces electrostatic repulsion between strands, resulting in a faster on-rate and a more stable hybridization among DNA molecules.

Thus, the strands are less likely to dissociate at higher salt concentrations and increased association rate. It is important to note that increasing the concentration of the complementary strands and higher salt concentrations influence the hybridization rates. As seen in the Figure 3(c), as expected the rate of association demonstrates a linear increase with the concentration of the complementary strand. Because the geometric arrangements and number of the docking strands are constant within the same experiment. The relationship between *κ*_on_ and concentration yields an on-rate constant of (2.79 ± 0.06) × 10^5^ (M s)^−1^ with a coefficient of correlation *R* ^2^: 0.99. However, the on-rate in the saline environment does not exhibit a clear linear relationship, as shown in Figure 3(f). After an initial increase, the estimated *κ*_on_ saturates with further concentration, followed by a sharp decline, as is seen at 339 nM. This behaviour might be due to the permanent occupation of docking strands at higher concentrations. The on-rate constant is (10.79 ± 0.4) × 10^5^ for 113 nM, (16.32 ± 0.5) × 10^5^ for 226 nM and (7.71 ± 0.4) × 10^5^ (M s)^−1^ for 339 nM. The observed *κ*_on_ value in a saline environment is higher than in water, leading to the enhancement in DNA hybridization.

Additionally, we further analysed the impact of ionic strength on the DNA hybridization kinetics. A comprehensive examination of the concentration dependence of dissociated and bound waiting times under various NaCl concentration (25, 50, 75, 100, 200, 300, 500, and 600 mM) in water, while the complementary strand concentration is maintained at approximately 113 nM was conducted. The measurements at each concentration step were performed over 20-min interval. The estimated *κ*_off_, plotted as black dots, and *κ*_on_, shown as a red triangle, depicted in Figure 3(i), exhibited distinctive rates for lower and higher salt concentrations. On-rate displayed a gradual increase at lower salt concentrations (25, 50, and 75 mM). However, a significant increase in *κ*_on_ was observed upon increasing the concentration to 100 mM, indicating a double increase in magnitude from (0.05 ± 0.02) s^−1^ at 75 mM to the (0.12 ± 0.05) s^−1^ at 100 mM. At low salt concentrations, the electrostatic repulsion between negatively charged DNA strands is not adequately shielded. This repulsion destabilizes the dsDNA, resulting in a higher dissociation rate and lower association rate as strands struggle to come together and stay paired. During the experiment, *κ*_off_ exhibits a contrasting trend by increasing the salt concentration as expected. Higher salt concentrations extend the duration of interactions due to the improved accessibility of the ssDNA to the docking strands. The situation is different for *κ*_off_, as its values sees a decrease from (15.49 ± 0.7) s^−1^ at 25 mM to (12.73 ± 0.2) s^−1^ at 75 mM. A more substantial decrease is observed at 100 mM, where *κ*_off_ is about (8.60 ± 0.1) s^−1^. Increased ionic strength effectively shields the negative charges, allowing strands to hybridize more efficiently. As shown, the decrease in *κ*_off_ is more significant at lower salt concentrations (25–75 mM) than at higher concentrations (100–300 mM) and can be attributed to the initial substantial shielding of electrostatic repulsion by salt. As the concentration of salt increases further, the effect of additional salt on reducing repulsion and stabilizing the dsDNA diminishes, leading to a gradual decrease in *κ*_off_.

When the salt concentration is increased from 500 to 600 mM, the on-rate values remain relatively constant, ranging from approximately (0.29 ± 0.02) to (0.30 ± 0.05) s^−1^. This indicates the onset of a plateau, suggesting that the system is approaching a saturation point. Consequently, further increases in salt concentration do not enhance hybridization rate. At this saturation point, docking strands have hybridized with complementary strands, as confirmed by steps in Δ*λ* shown in S5 and the hybridization rate is now limited by the availability of docking strands rather than complementary strands. Thus, further increases in the salt concentration do not significantly increase on-rate, as the docking strands are already engaged in hybridization. However, increasing the salt concentration can continue to stabilize the duplexes, possibly further reducing *κ*_off_.

## 6 Discussion

The integration of DNA origami structures with opto-plasmonic WGM sensors represents a substantial advancement in label-free (fluorescence-free) biosensing, overcoming limitations faced by traditional methods and offering improved sensitivity and specificity in detecting DNA hybridization events on DNA origami structures. The DNA origami structure serves as a scaffold for assembling a plasmonic nanorod dimer, enabling WGM sensors to detect DNA hybridization through resonance wavelength shifts. Plasmonic nanorod dimers significantly enhance detection sensitivity compared to monomers by generating stronger near-field enhancements in the nanogap between the nanorods, which is essential for detecting single-molecule events. However, the performance of these sensors can be affected by size and shape deviations of the AuNRs arising from fabrication limitations. Features such as rounded edges, surface roughness, and small deviations in the gap size between nanorods can lead to spectral detuning of the LSPR, altering the resonance position and field distribution near the nanogap. Such detuning can diminish field enhancement, impacting the sensor’s sensitivity and specificity. As Baaske et al. [30] have shown, even atomic-scale surface roughness can profoundly influence field enhancement and resonance shifts, highlighting the importance of precise fabrication techniques to ensure consistent sensor performance. In this study, we focus on the interactions occurring specifically within the nanogap, reducing the impact of AuNR size and shape variations compared to previous studies [31], [43], [44], where these structural parameters played a central role in signal amplitude. However, achieving consistent control over gap size remains challenging due to fabrication constraints and variability in the DNA structures used to assemble AuNRs. Additionally, the variability in the placement of DNA docking strands may further contribute to signal amplitude variability, as the strands may not always align within the region of optical field enhancement in the gap. Despite these challenges, our results demonstrate that reliable single-molecule detection can still be achieved, highlighting the robustness and versatility of the sensor. The use of DNA origami ensures precise assembly of the AuNR dimers, while maintaining a controlled gap size is essential for high-sensitivity sensing platforms. Moreover, the use of orthogonal (non-interfering) DNA sequences ensures that the binding site for the plasmonic nanoparticle does not interfere with the detection site, thereby enhancing the specificity and accuracy of the sensor. Orthogonal sequences also facilitate potential for multiplexing, enabling the simultaneous detection of multiple analytes without cross-reactivity, thereby turning the system into a more versatile and robust plasmonic platform for exploring a wide range of biomolecular sensing applications where single-molecule sensitivity and real-time detection are required. Our study thoroughly investigates the kinetics of DNA hybridization under different environmental conditions, specifically varying concentrations of complementary strands and NaCl. By showing these relationships, we contribute to a better understanding of the factors influencing DNA binding and unbinding dynamics, which is crucial for the development of more efficient and reliable DNA detection techniques.

The findings reveal that the salt concentration significantly influences the hybridization kinetics. Higher salt concentrations result in reduced electrostatic repulsion between the negatively charged DNA strands, thereby stabilizing the hybridized duplexes and prolonging the interactions between complementary and docking strands. This is evidenced by the decrease in dissociation rates and the increased association rates in saline environments compared to water. The observed linear relationship between on-rate and the concentration of complementary strands in water aligns with the expected behaviour, as reported in Refs. [31], [39], where increased concentration leads to more frequent hybridization events. The on-rate constant in water results in (2.79 ± 0.06) × 10^5^ (M s)^−1^. In contrast to previous reports [31], [43], which utilized a single AuNR as platform on the WGM with thiolated ssDNA docking strands immobilized on the AuNR surface, reported an on-rate constant in 500 mM NaCl of approximately (3.9 ± 0.5) × 10^4^ (M s)^−1^. This value is ten times lower than our observation, likely due to the differences in arrangement and accessibility of docking strands contributing to the signals on each sensor as well as the fact that only a few binding sites on the tips of the nanorods provide the required enhancement to detect single molecule events. Additionally, at a 300 mM NaCl concentration, the on-rate constant is (10.79 ± 0.4) × 10^5^ for a 113 nM complementary strand, which is more than twice the rate in water. As the complementary strand concentration increases, the association rate initially increases but eventually saturates and then declines at higher concentrations. This behavior might be due to the permanent binding of complementary strands at higher concentrations, resulting in the full occupation of docking strands and limiting further hybridization. The analysis of hybridization kinetics across a range of NaCl concentrations highlights the role of ionic strength in DNA interactions. At lower salt concentrations, high electrostatic repulsion results in higher dissociation rates and lower association rates. As the salt concentration increases, the shielding effect of sodium ions reduces this repulsion, enhancing the stability and formation of DNA duplexes. This effect plateaus around 500 mM NaCl, where docking strands have already hybridized with complementary strands so further increases in salt concentration do not significantly enhance hybridization rates but continue to stabilize the formed duplexes, potentially further reducing off-rate.

## Supporting information available

Microcavity fabrication; simulation of electric field enhancement within the dimer gap; TEM images of the DNA origami assembled AuNR dimers; the UV spectra functionalized AuNR, monomers and dimers; measuring the hybridization kinetics of DNA oligonucleotides using survivor function; step signal from DNA hybridization event at higher salt concentration; interaction of non-complementary strand with docking strand.

## Supplementary Material

Supplementary Material Details
