# Supplementary material for: Label-free (fluorescence-free) sensing of a single DNA molecule on DNA origami using a plasmon-enhanced WGM sensor
Source: Nanophotonics. 2025 Jan 20;14(2):253–62. doi: 10.1515/nanoph-2024-0560 (PMC11806501; doi:10.1515/nanoph-2024-0560)
Supplement: Supplementary file 1 — Supplementary Material Details [file j_nanoph-2024-0560_suppl_001.pdf]

Label-free (fluorescence-free) sensing of a single DNA molecule on DNA origami using a plasmon-enhanced WGM sensor

Shahin Ghamari[1†], Germán Chiarelli[2†], Karol Kołątaj[2,3], Sivaraman Subramanian[1], Guillermo P. Acuna[2,3*], Frank Vollmer[1*]

[1]Department of Physics and Astronomy, Living Systems Institute, University of Exeter, Exeter, EX4 4QD, UK

[2]Department of Physics, University of Fribourg, Chemin du Musée 3, Fribourg CH-1700, Switzerland.

[3]Swiss National Center for Competence in Research (NCCR) Bio-inspired Materials, University of Fribourg, Chemin des Verdiers 4, CH-1700 Fribourg, Switzerland.

[†]Equal Contribution

[*]f.vollmer@exeter.ac.uk, guillermo.acuna@unifr.ch

Supplementary Informations (SI)

SI1 Microcavity Fabrication

To fabricate a high-Q WGM microcavity (the fabrication setup shown in S1), a continuous 30 W CO_2 laser (Synrad 48-2, Novanta Inc., WA, USA) emitting at $\lambda = 10.6$ μm is employed to melt a single-mode optical fiber (SMF 28e, Corning GmbH, Germany). Following the removal of the protective coating layer, the fiber is cleaned with acetone and then isopropanol. One end of the fiber is secured with a small ceramic ferrule. After mounting the fiber with the ferrule on a manual stage, a small weight is attached to the free end of the fiber to keep tension. The fiber is then positioned at the focal point of the CO_2 laser. Subsequently, a stem with a width of about 10 μm and a length of 500 μm is made by tapering the fiber with 9.5-11% of the laser peak power. A glass sphere is formed at a slightly higher laser power of approximately 28-30% of peak power. The suspended stem-sphere is placed in a chamber enclosed by a V-shaped polydimethylsiloxane (PDMS) layer sandwiched between a coupling prism and a glass coverslip. Typically, microspheres within the range of $85 - 90$ μm in diameter are utilized in this study.

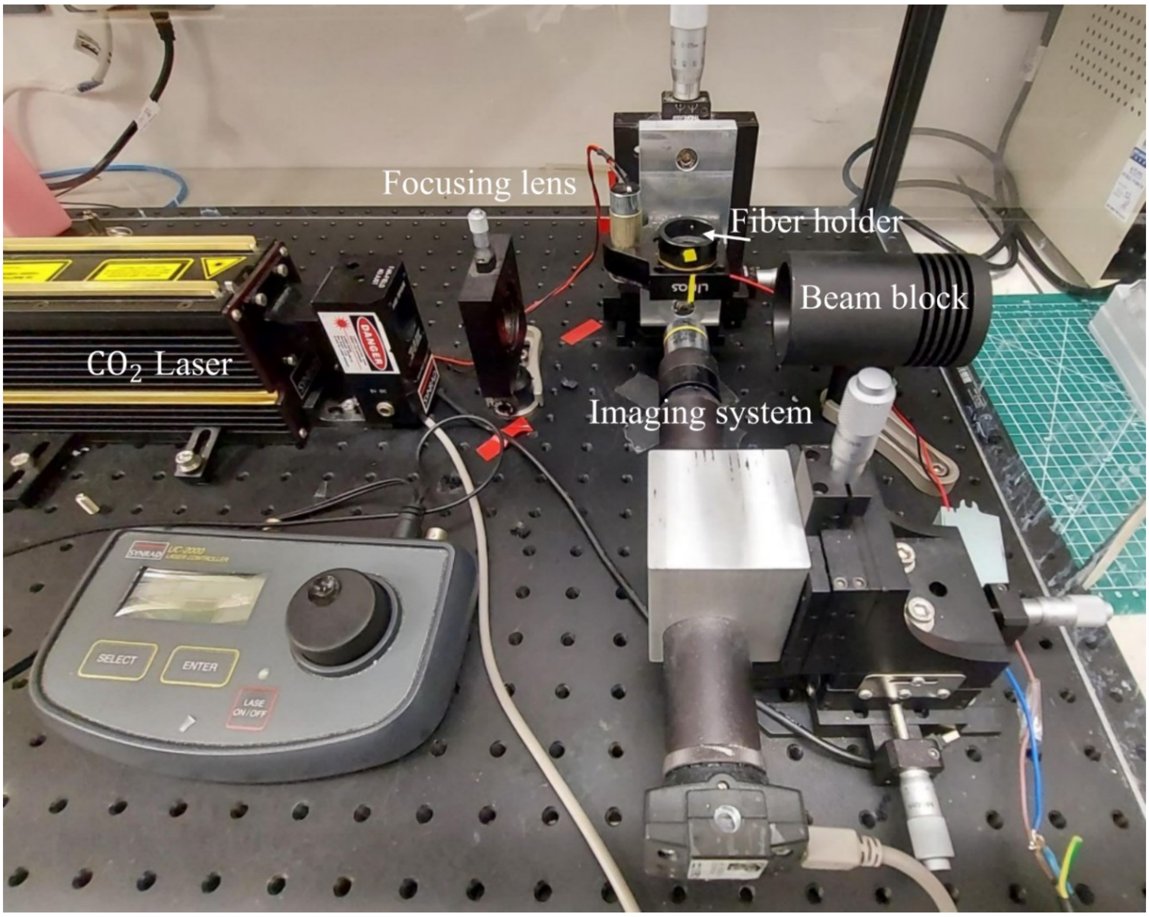

S1: The microresonator fabrication setup

SI2 Simulation of electric field enhancement within the dimer gap

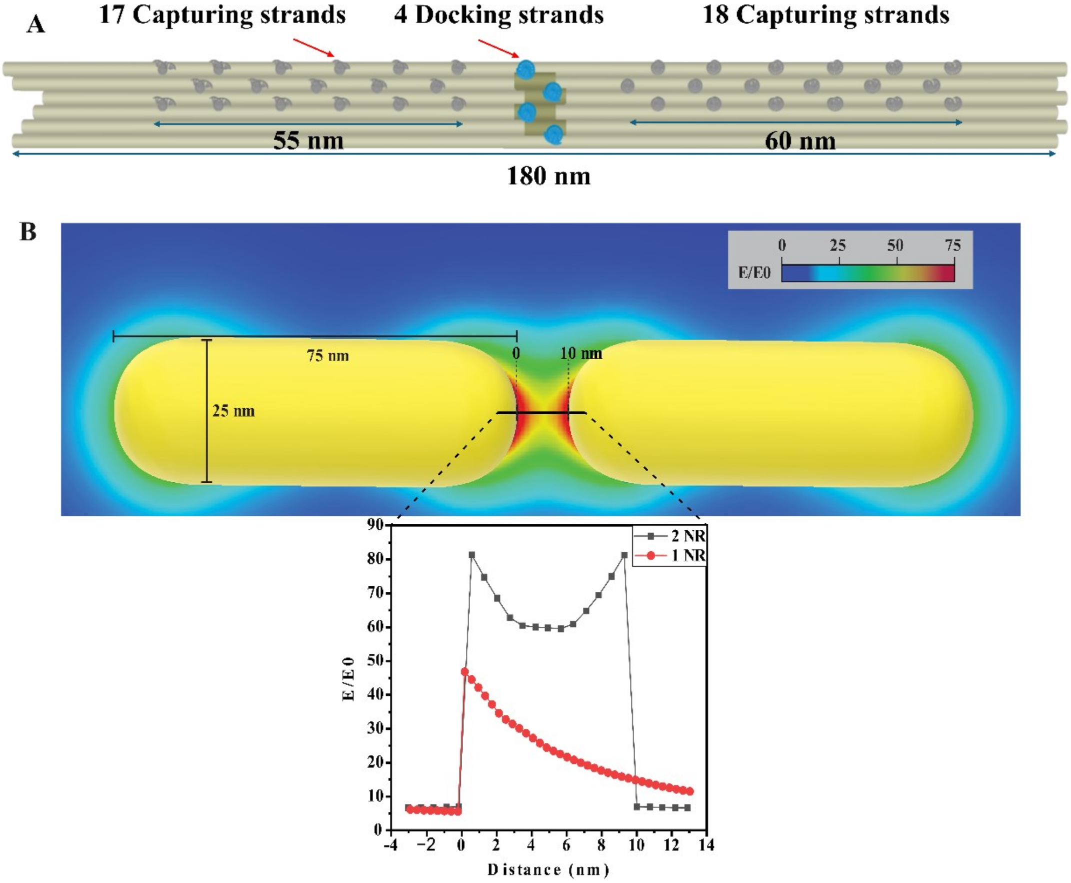

S2 A) presents the DNA origami modified by adding a total of 17 and 18 CSs at the left and right side of the structure with a mast at the center, where 4 DS are located, B) Simulations of LSPR were conducted to compare the electric field enhancement at the hotspot of a single AuNR and two aligned AuNR, both with dimensions of 25 × 75 nm. The electric field across a line spanning the 10 nm gap between the two AuNRs was analyzed, with the results depicted in the bottom graph. The distance along this line was measured from the end of the first AuNR, defined as 0 nm. At a point 5 nm away from the first AuNR, the electric field within the dimer was found to be 60 times stronger than the original field. In contrast, the monomer structure showed an enhancement of only 23 times.

SI3 TEM images of the DNA origami assembled AuNR dimers

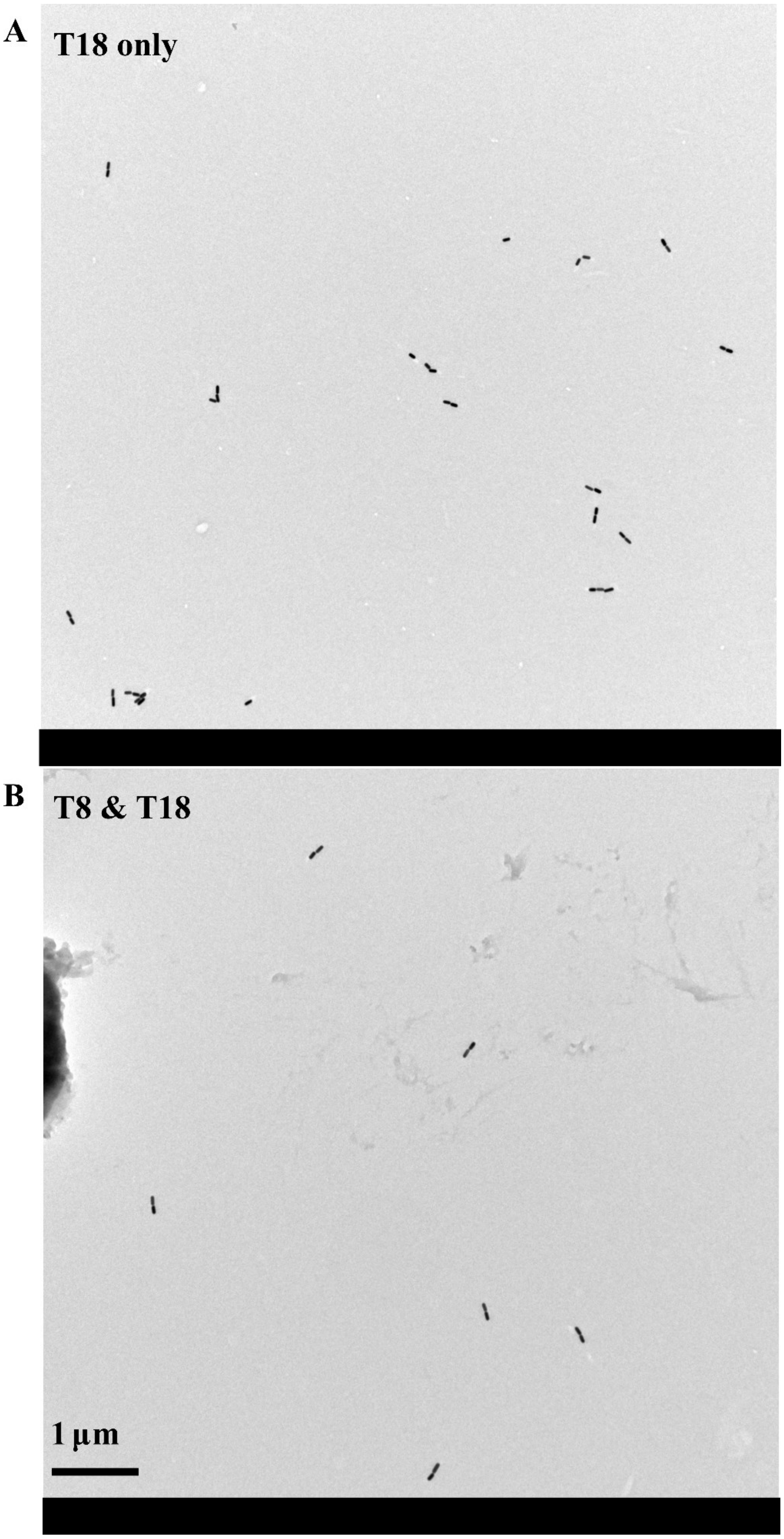

S3 The structures formed exclusively with T18 strands are shown in A), while B) highlights the dimers obtained through the combined use of T8 and T18 strands.

SI4 The UV spectra Functionalized AuNR, monomers and dimers

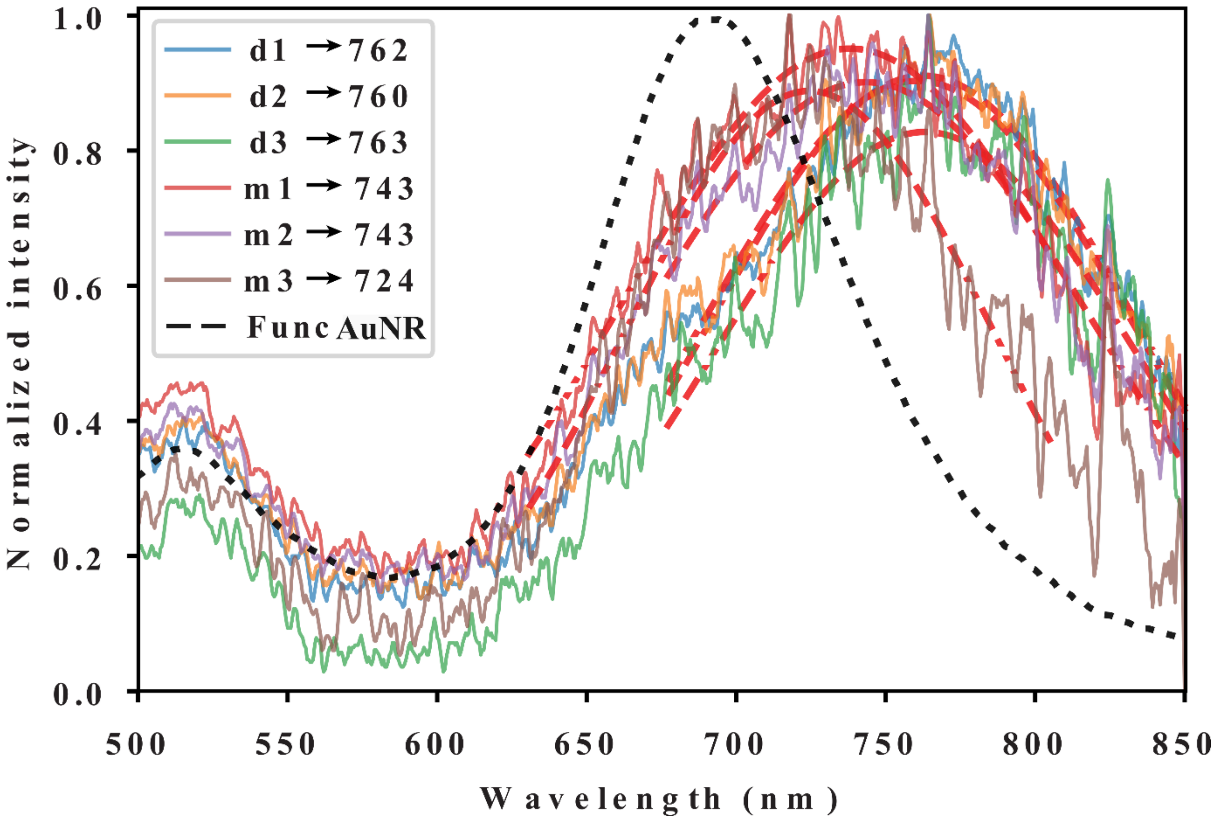

S4 The UV spectra of Func AuNR, monomers and dimers

SI5 Measuring the hybridization kinetics of DNA oligonucleotides using survivor function

By defining $N(T)$ as a number of hybridization events that has happened in the time interval T, $N(T)$ follows a Poisson distribution with a mean of RT, where R represents the rate of hybridization events. Thus, the probability distribution function (PDF) of observing N hybridization events in time interval T can be expressed mathematically as $P(N(T) = N) = \frac{(RT)^N}{N!} e^{-RT}$.

Following this, $P(T)_{on} = e^{-\kappa_{on}T}$ denotes the probability that no binding event has occurred within a given time interval T. In other words, it quantifies the likelihood of the system remaining in the non-binding state during this time. The parameter κ_{on} signifies the single-molecule binding rate, also recognized as the association rate, measured in s^{-1} and is directly proportional to Ac_i, where A is the on-rate constant with unit of $(M.s)^{-1}$ and c_i is the complementary strand concentration. The association rate is dependent on the concentration of the complementary strand and grows linearly as c_i increases. As freely diffusion complementary ssDNA engages in binding with the docking strand, the probability $P(t)_{off} = e^{-k_{off}t}$ that a dissociation event has not occurred within an interval t. This expression quantifies the probability that the bound state is maintained for the duration t without experiencing dissociation. Here k_{off} represents the single-molecule dissociation rate constant with unit of s^{-1}, and remains independent of the complementary strand concentration.

In our study, the available number of data points was relatively low, falling below 500 due to practical constraints. This limited dataset presented challenges for constructing meaningful histograms to visualize the probability distribution function (PDF), potentially affecting data smoothing and interpretation. As a result, we chose to employ the survivor function, offering valuable insights into survival probabilities without the need for extensive data visualization. Additionally, to obtain a larger dataset exceeding 500 data points, it would have been necessary to conduct experiments over an extended period. However, given time constraints, such prolonged experimentation was not feasible within the scope of our study. Thus, we opted to leverage the survivor function as an effective alternative for data analysis.

The survivor function, denoted as S(x), is defined as 1−F(x), where F(x) is the cumulative distribution function (CDF). In simpler terms, the survivor function represents the probability of surviving beyond a certain time t. Furthermore, the CDF is obtained by integrating the PDF over a given range.

SI6 Step signal from DNA hybridization event at higher salt concentration

The step signals are indication of the permanent binding of docking strands with complementary strands, as confirmed by steps in Δλ.

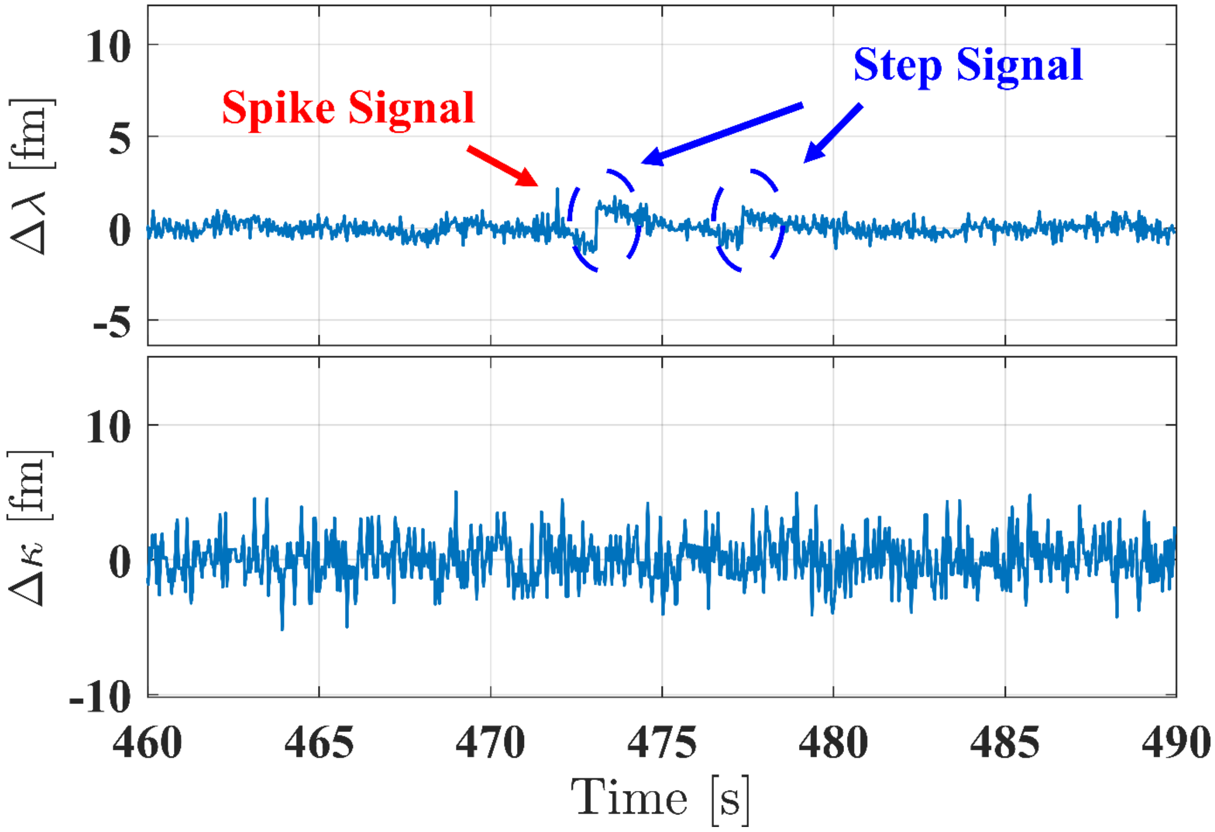

S5 Time traces of resonance shift for experiment with 600 mM NaCl and 113nM complementary strands concentration

SI7 Interaction of non-complementary strand with docking strand

To demonstrate the specificity of the DNA hybridization event between the docking and its complementary strands, a non-complementary strand with a concentration of 113 nM was introduced into the solution containing 500 mM NaCl. Figure S6 illustrates time traces of the WGM resonance wavelength shift, revealing no signal at various time points during this reaction.

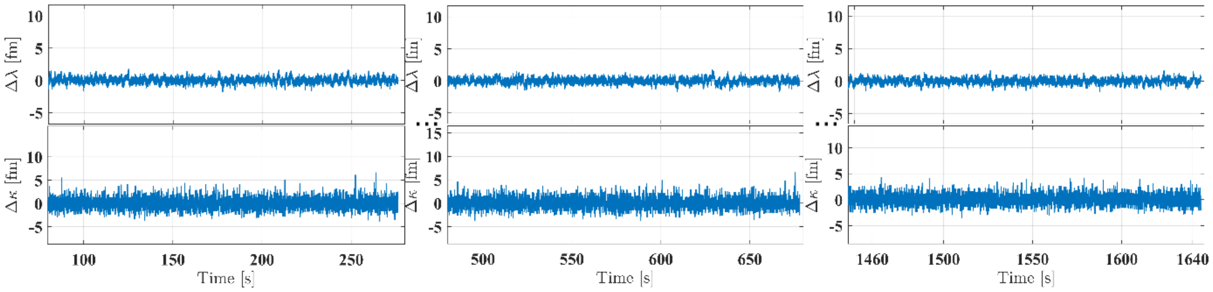

S6 Different Time traces of WGM resonance shift at different times
